# The WISHED Trial: implementation of an interactive health communication application for patients with chronic kidney disease

**DOI:** 10.1186/s40697-016-0120-y

**Published:** 2016-06-15

**Authors:** Andrea Harvey, Michael Walsh, Arsh K. Jain, Eric Bosch, Cathy Moreau, Jocelyn Garland, K. Scott Brimble

**Affiliations:** St. Michael’s Hospital, Toronto, ON Canada; Division of Nephrology, McMaster University, Hamilton, ON Canada; Division of Nephrology, University of Western Ontario, London, ON Canada; Division of Nephrology, Queen’s University, Kingston, ON Canada; McMaster University, 50 Charlton Ave. E, Hamilton, ON L8N 4A6 Canada

**Keywords:** Home dialysis, Interactive health communication application, Hemodialysis, Peritoneal dialysis, Internet

## Abstract

**Background:**

Despite many advantages over facility-based therapies, less than 25 % of prevalent dialysis patients in Ontario are on a home therapy. Interactive health communication applications, web-based packages for patients, have been shown to have a beneficial effect on knowledge, social support, self-efficacy, and behavioral and clinical outcomes but have not been evaluated in patients with chronic kidney disease (CKD). Web-based tools designed for patients with CKD exist but to our knowledge have not been assessed in their ability to influence dialysis modality decision-making.

**Objective:**

To determine if a web-based tool increases utilization of a home-based therapy in patients with CKD starting dialysis.

**Design:**

This is a multi-centered randomized controlled study.

**Setting:**

Participants will be recruited from sites in Canada.

**Participants:**

Two hundred and sixty-four consenting patients with an estimated glomerular filtration rate (eGFR) less than 20 ml/min/1.73 m^2^ who have received modality education will be enrolled in the study.

**Measurements:**

The primary outcome will be the proportion of participants who are on dialysis using a home-based therapy within 3 months of dialysis initiation. Secondary outcomes will include the proportion of patients intending to perform a home-based modality and measures of dialysis knowledge, decision conflict, and social support.

**Methods:**

The between-group differences in frequencies will be expressed as either absolute risk differences and/or by calculating the odds ratio and its associated 95 % confidence interval.

**Conclusions:**

This study will assess whether access to a website dedicated to supporting and promoting home-based dialysis therapies will increase the proportion of patients with CKD who initiate a home-based dialysis therapy.

**Trial registration:**

ClinicalTrials.gov #NCT01403454, registration date: July 21, 2011.

**Electronic supplementary material:**

The online version of this article (doi:10.1186/s40697-016-0120-y) contains supplementary material, which is available to authorized users.

## Abrégé

**Mise en contexte:**

L’administration de traitements d’hémodialyse à domicile présente plusieurs avantages par rapport aux traitements offerts en centre hospitalier. Pourtant, moins de 25 % des patients Ontariens suivent leurs traitements de dialyse à domicile. Bien que l’accès à des outils interactifs de communication en santé (OICS) ait des effets bénéfiques sur le niveau de connaissances, le soutien social, le niveau d’autonomie ainsi que sur les résultats cliniques et comportementaux des patients qui les utilisent, ces outils n’ont jamais fait l’objet d’études chez les patients atteints d’insuffisance rénale chronique (IRC). Des OICS existent pour cette population, mais on ne connaît pas leur part d’influence au moment où le patient doit faire le choix d’une technique de dialyse.

**Objectifs de l’étude:**

Par cette étude, on entend vérifier si l’accès à des outils sur le web augmentera le nombre de patients atteints d’IRC en amorce d’une dialyse qui choisiront d’effectuer leurs traitements à domicile.

**Cadre et type d’étude:**

Il s’agit d’un essai contrôlé, randomisé, qui se tiendra dans plusieurs centres hospitaliers à travers le Canada.

**Participants:**

La cohorte sera constituée de 264 patients atteints d’IRC dont le taux de filtration glomérulaire se situe à moins de 20 ml/min/1, 73 m^2^. Les participants auront suivi une séance d’orientation pour les aider à naviguer dans les différents outils mis à leur disposition sur le web.

**Mesures:**

À titre de résultat principal, on établira la proportion de patients qui auront adhéré à la technique de dialyse à domicile au cours des trois mois suivant l’initiation du traitement. On cherchera ensuite à connaître la proportion de patients ayant l’intention de le faire au courant de la première année de traitement. De plus, on procèdera à l’évaluation des connaissances et de la capacité des patients de prendre des décisions concernant leur traitement, ainsi que du soutien social qu’ils reçoivent.

**Méthodologie:**

Les différences entre les groupes d’étude seront exprimées soit sur le plan du risque absolu ou en calculant les rapports de cotes et les intervalles de confiance à 95 % correspondants.

**Conclusion:**

Cette étude évaluera si l’accès à un site web consacré au soutien social des patients et à la promotion des traitements de dialyse à domicile augmentera la proportion de patients souffrant d’IRC qui choisiront cette option pour l’amorce de leur traitement.

## What was known before

Interactive health communication applications are web-based tools that provide health information, social, decisional, and/or behavioral change support. A web-based tool that promotes the use of a home-based dialysis therapy has not been formally evaluated in patients with advanced chronic kidney disease (CKD).

## What this adds

This study will determine whether a web-based tool designed to promote home-based dialysis will increase its utilization in incident patients.

## Background

In a person-centered care model, patients with advanced chronic kidney disease (CKD) are provided with the necessary tools and information to select the type of dialysis therapy for which they are best suited. Many programs utilize a specially trained nurse to provide modality education to patients with more advanced CKD (i.e., estimated glomerular filtration rate [eGFR] less than 20 ml/min/1.73 m^2^). There are several modality choices for patients approaching end-stage renal disease (ESRD); these include peritoneal dialysis (PD), a home therapy, or hemodialysis (HD), which either can be at home (HHD) or performed in a facility. In Ontario, Canada, the provincial renal agency’s target for the home dialysis prevalence rate is 40 %, in contrast to the current provincial prevalence of 24 % [1].

Home dialysis offers many advantages over facility-based HD for patients. Home dialysis provides patients with scheduling flexibility which is rarely possible in facility-based HD. PD patients in particular have more freedom to travel and usually enjoy a less restrictive diet with respect to potassium than facility-based HD [2, 3]. Most observational studies suggest that home dialysis patients enjoy better scores in many quality of life domains, particularly treatment satisfaction and therapy intrusiveness [4–6], although some studies have not seen such a difference [7–9]. Home-based dialysis is also beneficial from the payer perspective; overall, healthcare costs are reduced by as much as US$20,000 per patient-year [10–12].

Numerous barriers to initiation of home-based therapies have been described, including provider beliefs, practices, and lack of adequate patient and provider education [13]. In addition to these systemic barriers to home dialysis, many barriers exist at the patient level, including lack of self-efficacy and confidence in performing the therapy, burden on family members, and fear of a catastrophic event [14–18]. Information gaps despite education being provided by care providers may lead to increased decisional uncertainty and conflict, particularly in an era when home-based therapies are being more actively encouraged. On the other hand, medical contraindication to a home therapy is uncommon; in one study, only 11 % of patients had a medical contraindication [18].

Interactive health communication applications (IHCAs) are computer-based packages for patients which are usually web-based and in addition to providing health information offer some form of social, decisional, and/or behavioral change support [19]. IHCAs facilitate the transfer of information and enable informed decision-making as well as the promotion of healthy behaviors and choices, peer information exchange and support, and self-care. A systematic review of IHCAs developed for individuals with chronic diseases such as diabetes mellitus (DM) and asthma identified 24 randomized controlled trials involving 3739 participants. IHCAs had a beneficial effect on knowledge, social support, self-efficacy, and behavioral and clinical outcomes [19]. Websites designed for patients with CKD who must make decisions regarding treatment options exist but to our knowledge have not been formally evaluated.

The primary objective therefore of this study is to determine if utilization of a website dedicated to the promotion of home-based dialysis will increase the proportion of patients who initiate dialysis using a home-based modality.

## Methods/design

### Study design and randomization

**Fig. 1:**
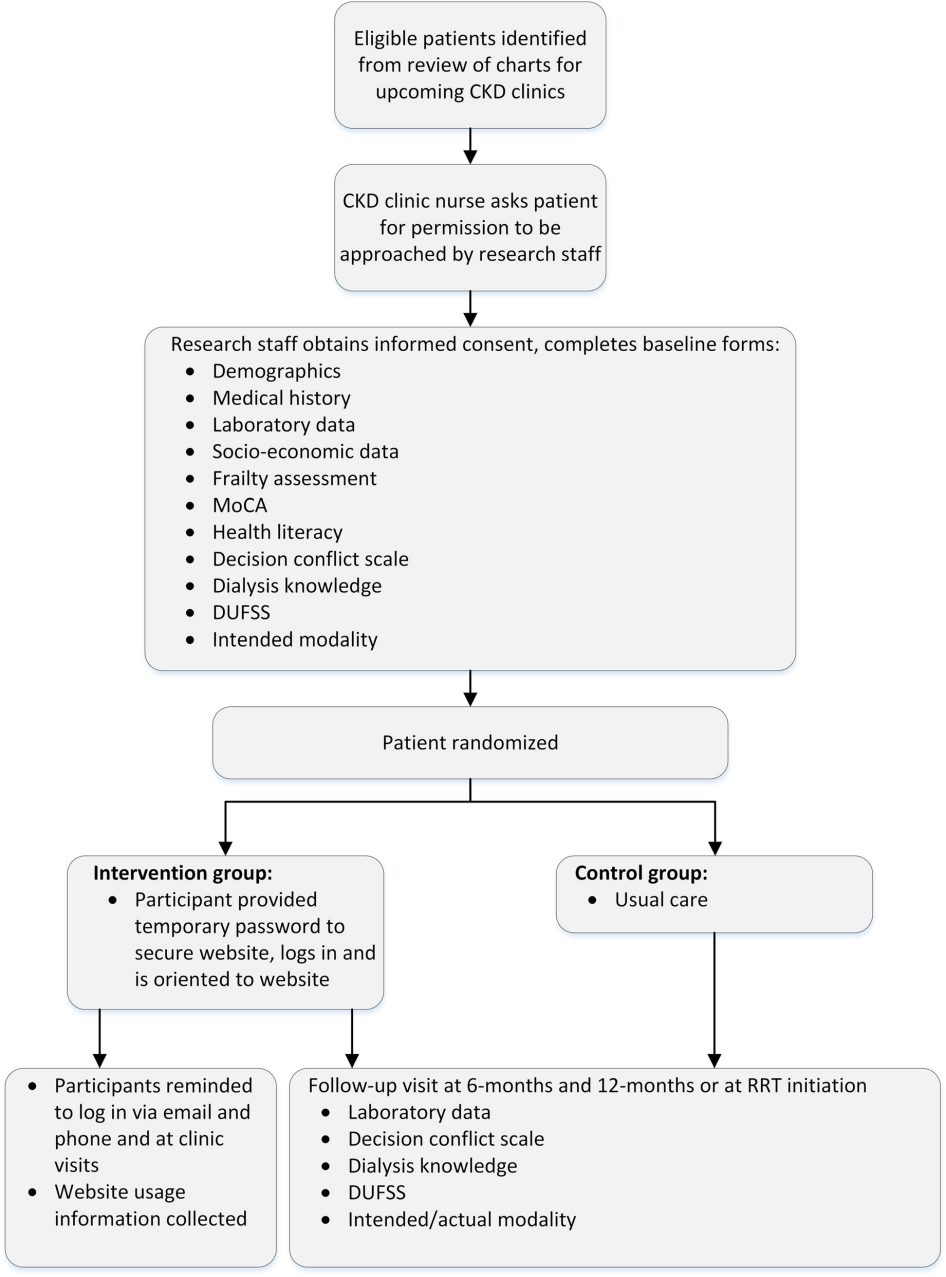
Flow of participants in the study

The study is a multi-centered randomized controlled trial comparing the use of a secured web-based IHCA (website www.independentdialysis.ca) versus usual care in the promotion of home-based dialysis therapies. A participant flow diagram is shown in Fig. 1. Randomization is performed using a computer-generated sequence in variable blocks, stratified by site and allocation occurring using sequentially numbered sealed opaque envelopes. Each study participant will be assigned a unique number. The information about randomization sequence and block size will be kept confidential.

### Setting

The intervention is currently taking place in three multidisciplinary CKD clinic sites in Ontario. Additional sites will be approached to participate as needed to achieve recruitment objectives. Each of the three sites is an academic regional referral center for patients for nephrology services including management of CKD, dialysis, and renal transplantation patients. There are currently about 2300 patients registered in multidisciplinary CKD clinics across the three sites. The study has been approved at each of the local institutional research ethics boards [Hamilton Integrated Research Ethics Board (HIREB)].

### Participants

All eligible participants are identified by the local CKD clinic electronic database and screened for eligibility by CKD nurses in the circle of care. All eligible participants are then approached and asked if they are interested in speaking to research staff about the study. Consented participants are then randomized into one of two study arms: (1) usual care or (2) the IHCA. The participant inclusion and exclusion criteria are shown in Table 1.

**Table 1:** Patient inclusion and exclusion criteria

Inclusion criteria	Exclusion criteria
1. Age ≥18 years 2. Enrolled in CKD clinic 3. Previously received ESRD modality education 4. Personal access to a home computer with Internet access 5. Most recent eGFR ≤20 ml/min/1.73 m^2^ 6. Declared intent for either dialysis or transplant	1. Absolute medical contraindication to home-based dialysis 2. Inability to provide informed consent 3. Inability to use a home computer and Internet 4. Inability to understand English (written and spoken) 5. Severe visual or auditory impairment

Abbreviations: *CKD* chronic kidney disease, *ESRD* end-stage renal disease, *eGFR* estimated glomerular filtration rate, *ml* milliliters, *min* minutes, and *m* ^*2*^ meter squared

### Intervention

Participants in both the usual care and IHCA arms continue to be seen in the CKD clinic as part of usual care. Participants randomized to the IHCA arm will be logged in to the website during the randomization visit and provided an orientation session to familiarize them with the website. They are asked to generate their own password and encouraged to log on to the website regularly. Email reminders to log-in are sent periodically and the frequency of participants’ visits monitored. The website was developed with a view to ensure easy navigation for participants, while providing content that encompasses informational and social support to reduce conflict and uncertainty in ESRD therapy decision-making. The informational support component of the website includes a section for *Frequently Asked Questions*, demonstration videos, and still photographs of equipment, as well as pre-recorded video interviews with local experts and existing patients. Updated information will continue to be added by a variety of content-expert healthcare professionals as it comes available. The social support component of the website will include video and text narratives of patients addressing the benefits and challenges of home dialysis, and a moderated forum for participants to discuss issues surrounding home dialysis with current home dialysis patients. Participants will also have the opportunity to email “experts,” including nephrologists, nurses, and existing home dialysis patients with any questions they may have. The available resources are available to all participants randomized to the intervention group, regardless of their intended modality.

### Outcomes

The primary outcome is the proportion of participants who receive any dialysis using a home-based therapy (PD or HHD) within 3 months of dialysis initiation. Participants that do not start dialysis or who receive a pre-emptive transplantation at study close will be regarded as non-home-based dialysis outcomes. Participants for which a modality cannot be ascertained will be considered non-home dialysis outcomes. Secondary outcomes include (1) proportion of patients intending to perform a home-based dialysis at 1 year, (2) dialysis knowledge as measured using a locally developed assessment tool (available online as Additional file 1), (3) decision conflict measured using the Decisional Conflict Scale [20], and (4) level of social support measured with the Duke-UNC Functional Social Support Questionnaire [21]. All of the above outcomes will be measured at baseline and 6 and 12 months post intervention.

### Statistical considerations

In all analyses, participants randomized will be analyzed according to the group to which they were allocated. All ratios and differences will be calculated as the experimental group compared to the control group. These analyses may be modified in the final statistical analysis plan at any time prior to the investigators accessing the study data in an unblinded fashion. The between-group differences in frequencies will be expressed as either absolute risk differences and their associated 95 % confidence intervals and/or by calculating the odds ratio and its associated 95 % confidence interval (exact binomial method). The between-group differences in continuous variables between groups will be assessed using repeated measures analyses. Sensitivity analyses will be performed by assessing the treatment effect after adjusting for baseline risk factors of known or highly suspected association with modality choice. These factors will include age, sex, diabetes mellitus status, socioeconomic strata, availability of a caregiver, presence of cognitive impairment (assessed by the Montreal Cognitive Assessment (MoCA) score [22], frailty [23], health literacy [24], and other comorbidities. To avoid model over-fitting, we will include only an appropriate number of variables (no more than one per 12 home-based dialysis events) and include them in the order in which they are written above. A two-sided *p* value of <0.05 will be regarded as significant without adjustment for multiple comparisons. The baseline probability of selecting home-based dialysis is assumed to be 28 % based on local data. Assuming an alpha value of 0.05, and 80 % power to detect a 22 % absolute difference between the intervention and usual care groups in the proportion of participants starting home-based dialysis, it is estimated that 152 participants would need to initiate dialysis to detect a significant difference between groups. Based on analysis of historical local clinic data on transition rates to ESRD therapies, it is anticipated that 264 participants will need to be recruited and followed for at least 1 year to achieve the required number of events. This recruitment target will be re-evaluated periodically and adjusted as needed based on differences between projected and when actual dialysis starts relative to the total number of recruited participants.

## Discussion

Patients with advanced CKD face what can be an overwhelming decision regarding their ultimate choice of dialysis modality. Home-based modalities are less costly and may provide a better quality of life for most patients. However, many Ontario programs are struggling to meet the provincial target of a 40 % home dialysis prevalence rate. Similar struggles have been noted in other jurisdictions. The objective of this intervention is to provide a supportive environment that is meant to encourage and support a decision to choose a home-based modality for patients with advanced CKD using a variety of methods, including informational, decisional, and social support utilizing an IHCA as a framework. This study will also evaluate whether such a tool has an effect on participants’ knowledge, sense of social support, and perceived decisional conflict. The findings from this study will help to inform whether such a tool would be effective in encouraging the use of home-based modalities in this population. The study will have the potential to expand on which, if any, baseline patient factors predict utilization of home-based modalities. From a payer perspective, more than 10,000 patients are on dialysis in Ontario of which the majority is using facility-based HD (>75 %). If this IHCA is an effective educational tool, this would result in improved patient outcomes and substantial healthcare cost-savings. The estimated 10-year provincial cost-savings if the home dialysis proportion increased to 40 % is over US$133 million. The IHCA will be a portable, easy to use, and inexpensive tool making it easily implementable across centers in Ontario and elsewhere.

In end-of-study knowledge translation, we intend to provide information and tools to promote the access and utilization of the website for all CKD programs in Canada. The tool will also be made available through the Kidney Foundation of Canada, the Canadian Society of Nephrology websites, and provincial agencies including the Ontario Renal Network.

## Additional file

Additional file 1: Dialysis knowledge survey. (DOCX 53 kb)

## Abbreviations

CKD: chronic kidney disease
eGFR: estimated glomerular filtration rate
HD: hemodialysis
HHD: home hemodialysis
IHCA: interactive health communication application
MoCA: Montreal Cognitive Assessment
PD: peritoneal dialysis
WISHED: web-based IHCA for successful home dialysis
